# *MUC4 *gene polymorphisms associate with endometriosis development and endometriosis-related infertility

**DOI:** 10.1186/1741-7015-9-19

**Published:** 2011-02-24

**Authors:** Cherry Yin-Yi Chang, Hui-Wen Chang, Chih-Mei Chen, Chia-Ying Lin, Chih-Ping Chen, Chih-Ho Lai, Wei-Yong Lin, Hsing-Ping Liu, Jim Jinn-Chyuan Sheu, Fuu-Jen Tsai

**Affiliations:** 1 Department of Obstetrics and Gynecology, China Medical University, Taichung, Taiwan; 2 Department of Public Health, China Medical University, Taichung, Taiwan; 3 Department of Pathology, China Medical University, Taichung, Taiwan; 4 Human Genetic Center, China Medical University Hospital, Taichung, Taiwan; 5 Department of Obstetrics and Gynecology, Mackay Memorial Hospital, Taipei, Taiwan; 6 School of Medicine, China Medical University, Taichung, Taiwan; 7 Graduate Institute of Integrated Medicine, China Medical University, Taichung, Taiwan; 8 Graduate Institute of Acupuncture Science, China Medical University, Taichung, Taiwan; 9 School of Chinese Medicine, China Medical University, Taichung, Taiwan; 10 Department of Health and Nutrition Biotechnology, Asia University, Taichung, Taiwan; 11 School of Post-Baccalaureate Chinese Medicine, China Medical University, Taichung, Taiwan

## Abstract

**Background:**

Mucin 4 (*MUC4*) plays an important role in protecting and lubricating the epithelial surface of reproductive tracts, but its role in the pathogenesis of endometriosis is largely unknown.

**Methods:**

To correlate *MUC4 *polymorphism with the risk of endometriosis and endometriosis-related infertility, we performed a case-control study of 140 patients and 150 healthy women. Six unique single-nucleotide polymorphisms (SNPs) (rs882605, rs1104760, rs2688513, rs2246901, rs2258447 and rs2291652) were selected for this study. DNA fragments containing the target SNP sites were amplified by polymerase chain reaction using the TaqMan SNP Genotyping Assay System to evaluate allele frequency and distribution of genotype in *MUC4 *polymorphisms.

**Results:**

Both the T/G genotype of rs882605 and the frequency of haplotype T-T (rs882605 and rs1104760) were higher in patients than in controls and were statistically significant. The frequency of the C allele at rs1104760, the C allele at rs2688513, the G allele at rs2246901 and the A allele at rs2258447 were associated with advanced stage of endometriosis. Moreover, the G allele at rs882605 was verified as a key genetic factor for infertility in patients. Protein sequence analysis indicated that amino acid substitutions by genetic variations at rs882605, rs2688513 and rs2246901 occur in the putative functional loops and the type D von Willebrand factor (VWFD) domain in the MUC4 sequence.

**Conclusions:**

*MUC4 *polymorphisms are associated with endometriosis development and endometriosis-related infertility in the Taiwanese population.

## Background

Endometriosis is a common chronic gynecological disease characterized by the presence of endometrial gland and stroma outside the uterine cavity, affecting approximately 10% of reproductive age women [1,2]. The common clinical symptoms include pelvic pain, heavy menstrual bleeding, pelvic adhesion, bloating and fatigue. Notably, the prevalence of endometriosis is 0.5% to 5% in fertile women and 25% to 40% in infertile women [3], suggesting infertility as one possible consequence of endometriosis. To date, the implantation theory is widely accepted, stating that endometrial tissues pass through the fallopian tube, then attach and grow on pelvic tissue. However, this hypothesis cannot explain the existence of endometriosis outside the pelvis or how endometriosis progresses and invades other tissues. Additional factors such as genetic or immune differences have been suggested as possible contributors to trigger the formation of endometriosis [4-6]. Family history and genome-wide linkage studies also support genetic predisposition during the development of endometriosis [7-10]. These studies provide molecular evidence demonstrating endometriosis as a genetic disease, and it is desirable to explore more genetic variations associated with endometriosis.

Similarly to malignant diseases, extensive growth of endometrial cells on the peritoneal surface and invasion of the pelvic organ are very common during the development of endometriosis. This process is frequently associated with several mechanisms involved in angiogenesis and cellular adhesion. In fact, women who have endometriosis appear to be more at risk of developing several different kinds of ovarian cancers [8,11-13]. An epidemiological study showed that the prevalence rates of endometriosis in patients with endometrioid and clear cell ovarian carcinoma are 19 and 35.9, respectively [14]. These findings suggest that endometriosis and certain types of ovarian cancer may share several common genetic alterations during pathogenesis. Genes that regulate cell mobility and invasion in ovarian cancers are therefore possible candidates for playing roles in endometriosis.

Mucins are high-molecular-weight glycoproteins with the function of protecting and lubricating the epithelial surface of respiratory, gastrointestinal and reproductive tracts [15]. Among the mucin proteins, mucin 4 (MUC4) and mucin 1 (MUC1) are the major ones expressed in the endometrial epithelium [16,17]. In cancer studies, these two mucins have been shown to be aberrantly expressed in various malignancies and have been validated as novel targets for cancer diagnosis and therapy [18-20]. Distinct from MUC1, the extracellular domain of MUC4 can interact with human epidermal growth factor receptor 2 (HER2) on the cell surface and modulate downstream cell growth signaling by stabilizing and/or enhancing the activity of cell growth receptor complexes [18,21,22]. Consequently, changes of cytoarchitectures and cellular signaling may lead to the increase of cell mobility and tumor cell invasion.

The above findings provide us with clues to hypothesize that genetic variations in the extracellular domain of MUC4, especially those resulting in amino acid substitutions, may play roles involved in the development of endometriosis. With endometriosis as a possible cause of infertility in women, we also would like to study the association of *MUC4 *single-nucleotide polymorphisms (SNPs) with susceptibility to endometriosis-related infertility.

## Methods

### Study population

A total of 140 individuals who underwent surgery for benign diseases and pathology-proven endometriosis were identified at China Medical University Hospital from 1998 to 2008 and were enrolled in this study. In general, these patients were diagnosed with ovarian cysts on the basis of sonography and had several clinical symptoms related to endometriosis, including dysmenorrhea, lower abdominal pain, infertility or abnormal menstruation. Study patients who failed to have pathology-proven endometriosis were excluded from this study. For the control group, blood samples of 150 healthy women were selected from a pool of individuals who received regular heath checkups at the same hospital and were identified as normal on the basis of the examinations conducted. A total of 142 controls were frequency-matched on the basis of age profile with the study patients (Additional file 1, Table S1). Controls who showed one of the endometriosis-associated symptoms, even though the results of their health checkups were normal, were excluded from this study. This study was approved by the institutional review board at China Medical University, with informed consent obtained from each patient.

### Clinical stages and association study

Clinical information on patients was collected from clinical notes, including clinical stage, lesion size, location, drug treatment and fertility (Additional file 1, Table S1). The definition of endometriosis staging was based on the classification of the American Society for Reproductive Medicine: Stage 1, minimal; Stage 2, mild; Stage 3, moderate; and Stage 4, severe [23].

### Genomic DNA extraction and genotyping of SNPs in *MUC4*

Genomic DNA was extracted from peripheral blood leukocytes according to standard protocols (Genomic DNA kit; Qiagen, Valencia, CA, USA). DNA fragments containing the target SNP sites were amplified by polymerase chain reaction (PCR) assay using the TaqMan SNP Genotyping Assay System from Applied Biosystems, Inc. (Carlsbad, CA, USA). The probe search and design are available on the Applied Biosystems, Inc. website [24].

Additional file 1, Table S2, lists probe identifications for the six SNPs tested. PCR amplification conditions consisted of initial denaturation at 95°C for 5 minutes followed by 40 cycles at 95°C for 10 seconds, 56°C for 10 seconds and 72°C for 20 seconds, with one additional cycle at 72°C for 5 minutes. Genetic variations were detected by reading the fluorescence signals of PCR products. A positive signal indicates a perfect match between the probe and the tested DNA, thus identifying the allele types. Ten percent of study participants were randomly chosen and genotyped in duplicate to confirm the concordance of the genotyping results. In our study, the call rates for these SNP probes were above 94% (Additional file 1, Table S3).

### Statistical analysis

The allelic frequency and genotype frequency distributions for the six polymorphisms of patients with endometriosis and the controls were performed by χ^2 ^analysis using SPSS software (version 10.0; SPSS Inc., Chicago, IL, USA). An unordered, 2 *df *two-sided test was used for the statistical analyses of our genotyping results. *P *< 0.05 was considered statistically significant. Allelic and genotypic frequencies are expressed as percentages of total alleles and genotypes. Odds ratios (ORs) were calculated from allelic and genotype frequencies with 95% confident intervals (95% CIs). The major (also the wild-type) allele was used as the reference for the allelic analyses. For the genotypic analyses, the homozygous major allele genotype was used as the referent group. Adherence to the Hardy-Weinberg equilibrium (HWE) constant was tested using a χ^2 ^test with 1 *df*. To study the association of the six SNPs with clinical stages and reproductive ability, Fisher's exact tests instead of χ^2 ^tests were used because of the small number of participants tested.

The haplotypes of each individual were determined using the Bayesian statistical method available in the free download software program PHASE 2.1 [25]. This approach incorporates *a priori *expectations of haplotypic structure based on population genetics and coalescence theory. Lewontin's D' (|D'|) and the linkage disequilibrium (LD) coefficient *r*^2 ^were determined between selected pairs of biallelic loci [26]. Haploview version 3.2 software (Whitehead Institute for Biomedical Research, Cambridge, MA, USA) was used to examine the structure of the LD block [27]. This program uses two-marker expectation maximization to estimate the maximum likelihood values of the four gamete frequencies from which the D' and log of odds (LOD) values are derived. The genetic effects of the inferred haplotypes were analyzed in the same way as the analysis of polymorphisms. The reported haplotype percentages are estimated on the basis of allele frequencies and LD. The *P *values are based on a comparison of a given haplotype with all other haplotypes combined.

### Functional analyses and secondary structure predictions of MUC4 protein

Functional characterization and annotation of MUC4 were performed by aligning the sequence with functional motifs and/or signatures in the PROSITE protein domain database [28]. To predict the secondary structure of the MUC4 sequence, the Chou and Fasman method was used [29]. An improved method was applied to increase the accuracy of the predictions by locating nucleation regions with refined wavelet transformation technology and by calculating propensity factors with larger data sets [30]. The program gives the propensity of each residue to be a part of an α-helix, a β-strand or a loop. We considered propensities *P*_α _> 1.03 as significant for helices and propensities *P*_β _> 1.05 as significant for strands. Predicted regions with fewer than four contiguous residues were not considered secondary structure units. For a region with both helix and strand tendencies, the secondary structure conformed with higher propensity: *P*_α _>*P*_β _or *P*_β _>*P*_α _is predicted. To plot hydrophobicity and surface probability, the Kyte and Doolittle method [31] and Emini *et al*. surface accessibility prediction (SAP) [32] were used, respectively. We slid a window along the MUC4 sequence to assign a "hydrophobicity" or "surface probability" value to each amino acid. The values were summed in the window, and the results were plotted.

## Results

### *MUC4 *gene polymorphisms and endometriosis

**Figure 1:**
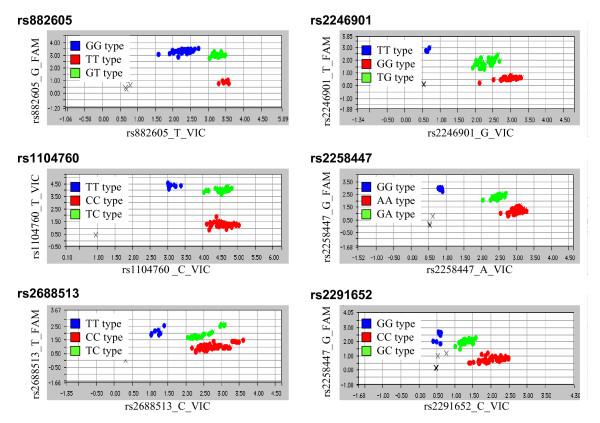
**Allelic discrimination plots of the six tested single-nucleotide polymorphisms (SNPs) in the mucin 4 (*MUC4*) gene**. The DNA samples from patients and controls were genotyped by using the TaqMan SNP Genotyping Assay System. The major (also the wild-type) alleles were detected by 6-carboxyfluorescein (FAM)-labeled probes (blue), and the minor alleles were detected by 2'-chloro-7'-phenyl-1,4-dichloro-6-carboxyfluorescein (VIC)-labeled probes. The genotyping results of the six SNPs in the *MUC4 *gene are presented as allelic discrimination plots. Of note, the intensity of FAM signals tended to be similar among samples in our assays, thus the dots for a wild-type genotype overlapped each other. "X" indicates the participant who failed to be genotyped.

Six SNPs in the extracellular domain of the *MUC4 *gene with a frequency greater than 20% in Chinese Han Beijing were selected from International HapMap Project databank [33] (Additional file 1, Table S2). Genotypic analyses (Figure 1) indicated rs882605 as a unique SNP with a higher frequency of the TG genotype in patients than in controls (*P *= 0.04, OR = 1.97, 95% CI, 1.17 to 3.32) (Table 1), while allele type analyses of these SNPs showed no statistical significance. Of note, the major (also the wild-type) allele was used as the reference for the allelic analyses. For the genotypic analyses, the homozygous major allele genotype was used as the referent group. To confirm the genetic impact of SNPs on endometriosis, the top two high-risk alleles at rs882605 and rs1104760 were selected for haplotype analyses. Significantly, the frequency of haplotype T-T was found to be higher in patients than in controls (*P *= 0.0353) (Table 2) (Additional file 2, Figure S1), suggesting the association of *MUC4 *SNPs with endometriosis development. The genotyping results were confirmed in duplicate, and the concordance of duplicates was 97.6%.

**Table 1:** Genotype and allele distributions of the six SNPs in the *MUC4 *gene in Taiwanese endometriosis patients and controls^a^

SNPs	Genotype/allele	No. (%)of patients		HWE	No. (%)of controls		HWE	*P *value	OR (95% CI)	
rs882605	TT	9	(7.0)	0.62	10	(7.2)	0.07	0.04	1.13	(0.43 to 3.00)
	TG	54	(42.2)		40	(28.8)			1.97	(1.17 to 3.32)^b^
	GG	65	(50.8)		89	(64.0)			1.00	Reference
	T	72	(28.1)		60	(21.6)		0.08	1.42	(0.96 to 2.11)
	G	184	(71.9)		218	(78.4)			1.00	Reference
rs1104760	CC	7	(5.4)	0.94	9	(6.3)	0.09	0.30	0.96	(0.33 to 2.82)
	CT	47	(36.2)		39	(27.5)			1.49	(0.89 to 2.51)
	TT	76	(58.5)		94	(66.2)			1.00	Reference
	C	61	(23.5)		57	(20.1)		0.34	1.22	(0.81 to 1.84)
	T	199	(76.5)		227	(79.9)			1.00	Reference
rs2246901	GG	8	(6.0)	0.96	10	(7.1)	0.15	0.56	0.91	(0.33 to 2.53)
	TG	49	(36.6)		43	(30.5)			1.30	(0.78 to 2.17)
	TT	77	(57.5)		88	(62.4)			1.00	reference
	G	65	(24.3)		63	(22.3)		0.60	1.11	(0.75 to 1.65)
	T	203	(75.7)		219	(77.7)			1.00	Reference
rs2258447	AA	9	(6.4)	0.37	10	(7.0)	0.07	0.70	1.02	(0.38 to 2.77)
	AG	44	(32.1)		40	(28.2)			1.25	(0.74 to 2.11)
	GG	81	(61.4)		92	(64.8)			1.00	Reference
	A	62	(22.5)		60	(21.1)		0.57	1.12	(0.75 to 1.68)
	G	206	(77.5)		224	(78.9)			1.00	Reference
rs2291652	CC	11	(8.7)	0.92	11	(8.4)	0.26	0.52	1.17	(0.46 to 2.96)
	CT	52	(40.9)		45	(34.4)			1.35	(0.81 to 2.28)
	TT	64	(50.4)		75	(57.3)			1.00	Reference
	C	74	(29.1)		67	(25.6)		0.36	1.20	(0.81 to 1.76)
	T	180	(70.9)		195	(74.4)			1.00	Reference
rs2688513	CC	8	(6.0)	0.62	10	(7.0)	0.09	0.66	0.91	(0.33 to 2.53)
	TC	45	(33.8)		41	(28.9)			1.25	(0.74 to 2.10)
	TT	80	(60.2)		91	(64.1)			1.00	Reference
	C	61	(22.9)		61	(21.5)		0.68	1.09	(0.73 to 1.63)
	T	205	(77.1)		223	(78.5)			1.00	Reference

^a^*MUC4*, mucin 4 gene; SNP, single-nucleotide polymorphism; OR, odds ratio; 95% CI, 95% confidence interval; HWE, *P *values of deviation from the Hardy-Weinberg equilibrium constant. Allelic frequencies were determined by χ^2 ^test using 2 × 2 contingency tables. Genotype frequencies were determined by χ^2 ^test using 2 × 3 contingency tables. ^b^*P *< 0.05 was considered statistically significant.

**Table 2:** Haplotype frequencies of *MUC4 *polymorphisms in endometriosis patients and controls^a^

rs1104760/rs882605	Patients, %	Controls, %	*P *value
TG	73.2	78.8	0.1202
CT	22.9	20.1	0.4134
TT	3.9	1.1	0.0353^b^

^a^*MUC4*, mucin 4 gene. The reported haplotype percents are estimated percents based on allele frequencies and the linkage disequilibrium. The *P *values are based on a comparison of a given haplotype with all other haplotypes combined. ^b^*P *< 0.05 was considered statistically significant.

### Association of *MUC4 *gene polymorphisms and stages

We next asked whether *MUC4 *genetic variations could possibly associate with clinical stages. Patients were divided into two groups: the mild stage group with patients at stages 1 or 2 and the advanced group with patients at stages 3 or 4. Strikingly, genotype analyses revealed strong associations of CC type at rs2688513 (*P *= 0.04) and GG type at rs2246901 (*P *= 0.03), with more advanced endometriosis at stage 3 or 4 (Table 3). Dominant effects were found for other genetic variations at rs1104760 (CC + CT versus TT) and rs2258447 (AA + AG versus GG) during endometriosis progression. Allele type analyses suggested C allele at rs1104760, C allele at rs2688513, G allele at rs2246901 and A allele at rs2258447 as risk factors that correlated with more severe endometriosis.

**Table 3:** Genotype and allele distributions of SNPs in the *MUC4 *gene in endometriosis patients at different clinical stages^a^

SNP	Genotype/allele	**No. (%) at mild stage**^**b**^		**No. (%) at severe stage**^**c**^		*P *value
rs882605	TT	0	(0.0)	7	(8.0)	0.25
	TG	2	(25.0)	41	(46.6)	
	GG	6	(75.0)	40	(45.5)	
	TT+TG	2	(25.0)	48	(54.5)	0.11
	GG	6	(75.0)	40	(45.5)	
	T	2	(12.5)	55	(31.3)	0.12
	G	14	(87.5)	121	(68.8)	
						
rs1104760	CC	0	(0.0)	5	(5.8)	0.05
	CT	0	(0.0)	36	(41.9)	
	TT	7	(100.0)	45	(52.3)	
	CC+CT	0	(0.0)	41	(47.7)	0.0^d^
	TT	7	(100.0)	45	(52.3)	
	C	0	(0.0)	46	(26.7)	0.03^d^
	T	14	(100.0)	126	(73.3)	
						
rs2688513	CC	0	(0.0)	6	(6.9)	0.04^d^
	CT	0	(0.0)	34	(39.1)	
	TT	8	(100.0)	47	(54.0)	
	CC+CT	0	(0.0)	40	(46.0)	0.01^d^
	TT	8	(100.0)	47	(54.0)	
	C	0	(0.0)	46	(26.4)	0.02^d^
	T	16	(100.0)	128	(73.6)	
						
rs2246901	GG	0	(0.0)	6	(6.8)	0.03^d^
	TG	0	(0.0)	36	(40.9)	
	TT	8	(100.0)	46	(52.3)	
	GG+TG	0	(0.0)	42	(47.7)	0.01^d^
	TT	8	(100.0)	46	(52.3)	
	G	0	(0.0)	48	(27.3)	0.02^d^
	T	16	(100.0)	128	(72.7)	
						
rs2258447	AA	0	(0.0)	7	(7.9)	0.08
	AG	0	(0.0)	32	(36.0)	
	GG	7	(100.0)	50	(56.2)	
	AA+AG	0	(0.0)	39	(43.8)	0.02^d^
	GG	7	(100.0)	50	(56.2)	
	A	0	(0.0)	46	(25.8)	0.03^d^
	G	14	(100.0)	132	(74.2)	
						
rs2291652	CC	0	(0.0)	7	(8.3)	0.61
	CT	2	(33.3)	36	(42.9)	
	TT	4	(66.7)	41	(48.8)	
	CC+CT	2	(33.3)	43	(51.2)	0.40
	TT	4	(66.7)	41	(48.8)	
	C	2	(16.7)	50	(29.8)	0.33
	T	10	(83.3)	118	(70.2)	

^a^SNP, single-nucleotide polymorphism; *MUC4*, mucin 4 gene. Allelic frequencies were determined by Fisher's exact test using 2 × 2 contingency tables. Genotype frequencies were determined by Fisher's exact test using 2 × 3 contingency tables. ^b^Mild stage, patients at clinical stage1 or stage 2; ^c^Severe stage, patients at clinical stage 3 or stage 4; ^d^*P *< 0.05 was considered statistically significant.

### *MUC4 *gene polymorphisms and infertility

Since endometriosis has been suspected as one potent factor leading to infertility in women [3], we also studied the possible linkage between *MUC4 *SNPs and infertility. Though no significant difference was found in our genotype association study, our data indicated T allele at rs882605 as a protective factor that associated with reduced frequency of infertility in patients with endometriosis (Table 4). Two other alleles, C at rs2688513 and G at rs2246901, showed similar protective effects, but the data did not reach statistical significance. Of note, the major (also the wild-type) allele was used as the reference for the allelic analyses. For the genotypic analyses, the homozygous major allele genotype was used as the referent group. In our haplotype analyses, we thus sought to ascertain the impact of the genetic combination of these top three protective alleles. Table 5 indicates that patients with haplotype T-C-G did show a lower frequency of infertility, although the results did not show a statistically significant difference (*P *= 0.099). By contrast, haplotype G-T-T showed a strong association with infertility in patients (*P *= 0.012) (Table 5) (Additional file 2, Figure S2) and could be used as a risk indicator for patients at higher risk of developing severe complications such as infertility.

**Table 4:** Genotype and allele distributions of the six SNPs in the *MUC4 *gene in endometriosis patients with different reproductive ability^a^

SNP	Genotype/allele	No. (%) infertility		No. (%) noninfertility		*P *value	OR (95% CI)	
rs882605	TT	0	(0.0)	8	(8.7)	0.07	-	-
	TG	5	(26.3)	42	(45.7)		0.36	(0.12 to 1.08)
	GG	14	(73.7)	42	(45.7)		1.00	Reference
	T	5	(13.2)	58	(31.5)	0.03^b^	0.33	(0.12 to 0.89)
	G	33	(86.8)	126	(68.5)		1.00	Reference
rs1104760	CC	0	(0.0)	6	(6.3)	0.41	-	-
	CT	4	(25.0)	37	(38.5)		0.48	(0.14 to 1.60)
	TT	12	(75.0)	53	(55.2)		1.00	Reference
	C	4	(12.5)	49	(25.5)	0.12	0.42	(0.14 to 1.25)
	T	28	(87.5)	143	(74.5)		1.00	Reference
rs2688513	CC	0	(0.0)	7	(7.4)	0.32	-	-
	TC	5	(25.0)	34	(36.2)		0.52	(0.17 to 1.56)
	TT	15	(75.0)	53	(56.4)		1.00	Reference
	C	5	(12.5)	48	(25.5)	0.09	0.42	(0.15 to 1.12)
	T	35	(87.5)	140	(74.5)		1.00	Reference
rs2246901	GG	0	(0.0)	7	(7.4)	0.25	-	-
	TG	5	(25.0)	37	(38.9)		0.46	(0.15 to 1.38)
	TT	15	(75.0)	51	(53.7)		1.00	Reference
	G	5	(12.5)	51	(26.8)	0.06	0.39	(0.14 to 1.05)
	T	35	(87.5)	139	(73.2)		1.00	Reference
rs2258447	AA	1	(5.0)	7	(7.3)	0.41	0.53	(0.05 to 5.53)
	AG	4	(20.0)	33	(34.4)		0.45	(0.14 to 1.48)
	GG	15	(75.0)	56	(58.3)		1.00	Reference
	A	6	(15.0)	47	(24.5)	0.22	0.54	(0.22 to 1.38)
	G	34	(85.0)	145	(75.5)		1.00	Reference
rs2291652	CC	0	(0.0)	7	(7.8)	0.29	-	-
	CT	7	(35.0)	40	(44.4)		0.58	(0.21 to 1.60)
	TT	13	(65.0)	43	(47.8)		1.00	Reference
	C	7	(17.5)	54	(30.0)	0.12	0.49	(0.21 to 1.19)
	T	33	(82.5)	126	(70.0)		1.00	Reference

^a^SNP, single-nucleotide polymorphism; *MUC4*, mucin 4 gene; OR, odds ratio; 95% CI, 95% confidence interval. Allelic frequencies were determined by Fisher's exact test using 2 × 2 contingency tables. Genotype frequencies were determined by Fisher's exact test using 2 × 3 contingency tables. ^b^*P *< 0.05 was considered statistically significant.

**Table 5:** Haplotype frequencies of *MUC4 *polymorphisms in endometriosis patients with infertility^a^

rs882605/rs2246901/rs2688513	Infertility, %	Noninfertility, %	*P *value
GTT	87.2	67.4	0.012^b^
TGC	12.5	24.4	0.099
TTT	0.2	4.4	0.206
GGT	0.0	2.2	0.344

^a^*MUC4*, mucin 4 gene. The reported haplotype percentages are estimated percentages based on allele frequencies and the linkage disequilibrium. The *P *values are based on a comparison of a given haplotype with all other haplotypes combined. ^b^*P *< 0.05 was considered statistically significant.

### *MUC4 *gene polymorphisms and amino acid substitutions

**Figure 2:**
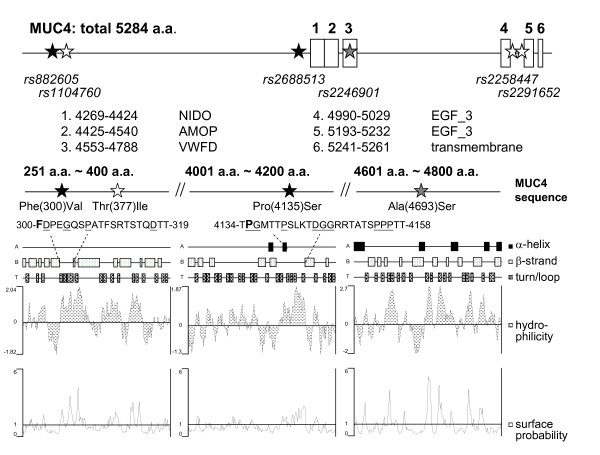
**Functional domains in the MUC4 protein sequence and the predicted secondary structures**. Six functional domains and/or signatures (boxes) were annotated by aligning MUC4 protein sequence in PROSITE protein domain database [28]. The boundaries of each signature are listed. Among six SNPs tested in this study (stars), rs882605 and rs2688513 (black) were found in two different long-loop regions, 300-319 and 4,134-4,158, respectively (bold letters refer to amino acid substitution sites). The SNP rs2246901 (gray) was found in a type D von Willebrand factor (VWFD) domain. The *MUC4 *reference sequence can be located at National Center for Biotechnology Information databank NP_060876.4.

Because these endometriosis-associated SNPs can cause amino acid substitutions (Additional file 1, Table S2), the biofunctions of MUC4 might be altered by changes in hydrophilicity and protein folding. Figure 2 illustrates the functional domains in MUC4 protein sequence and secondary structures that contain these SNPs. Our data show that genetic variations of rs882605 and rs2688513 cause amino acid substitutions in long-loop regions (> 10 residues) between secondary structure units (α-helices or β-strands) with high hydrophilicity and moderate surface probability (Figure 2). Amino acid composition analyses also revealed that these two loops (300-319 and 4,134-4,158) contain several negatively charged aspartate (D) residues and reverse turn elements, glycine (G) and proline (P), suggesting the importance of these loops in protein folding and functional regulation [34,35]. In addition, rs2246901 locates in a type D von Willebrand factor (VWFD) domain responsible for protein-protein interaction and cell adhesion and/or migration [36,37]. Our findings support the functional roles of *MUC4 *SNPs in regulating cellular mobility and invasion of endometrial cells during endometriosis development and progression.

## Discussion

Previous studies have shown polymorphism of cytokines and adhesion molecules which were associated with the pathogenesis of endometriosis [38,39]. To the best of our knowledge, however, no other study to date has investigated the possible association of *MUC4 *and endometriosis. The purpose of this study was to evaluate whether genetic variations in *MUC4 *associate with endometriosis in the Taiwanese population. Our data prove the association of *MUC4 *polymorphisms with advanced stages of endometriosis and the related infertility. Since the extracellular domain of MUC4 is critical for HER2 interaction and cell invasiveness, these defined SNPs located in putative functional domains of MUC4 may play important roles during endometriosis development and progression.

The development of endometriosis and certain types of ovarian cancer share several similar clinical features. For example, endometriosis could progressively invade pelvic viscera, resulting in adhesion, and could recur after medical treatment or surgery. Because of the functions involved in the acquisition of adhesion ligands or receptors and the loss of antiadhesion, proteins such as MUC4 and MUC1, the two major mucins present in endometrial epithelium, thus become suspect in endometriosis development [16,17]. In addition to gene overexpression, genetic variations in *MUC1 *have also been reported as risk factors contributing to cell mobility and the severity of cancer [40-43]. However, the influence of *MUC4 *genetic variations on cell behavior remains unclear. In this study, Pro4135Ser (rs2688513) and Ala4693Ser (2246901) substitutions in the putative functional domains of MUC4 were found to be associated with advanced stages of endometriosis. Since MUC4 is an emerging target for ovarian cancer [18,19,44,45], our study provides a new direction from which to address the roles of MUC4 in the development of gynecological disorders.

To study the genetic effects of mucin proteins by SNPs, the major interest focuses on the variable number of tandem repeat (VNTR) polymorphisms, which result in different-sized gene transcripts. For examples, *MUC1 *variations in the VNTR domain have been found to play roles in regulating *Helicobacter pylori *binding to gastric cells [46]. Other studies have also concluded that VNTR polymorphisms can influence T-antigen presentation and the local immune responses, which consequently have potential effects on gastric cancer development [47,48]. With regard to *MUC4*, a high degree of polymorphism in the VNTR domain was observed in human tissues, including the endometrial epithelium [49,50]. However, the different-sized *MUC4 *transcripts did not show association with embryo implantation or cancer development. By contrast, MUC4 can promote cell proliferation and antiapoptotic effects in cancer cells by interacting with HER2 on the cell surface [18,19,22], suggesting the potency of functional domains in the extracellular domain of MUC4. In this study, two SNPs (rs2688513 and rs2246901) that locate in a putative functional loop and the VWFD protein binding domain, respectively, were found to be associated with advanced stages of endometriosis. Further study may clarify whether these amino acid substitutions could change the interaction with HER2 and/or play crucial roles in regulating the cellular activity of the spread of endometriosis.

Endometriosis could cause pelvic adhesion and tubal occlusion that may lead to infertility. However, among patients with endometriosis-related infertility, 50% to 60% of them were diagnosed with minimal or mild endometriosis [3]. Impaired folliculogenesis, bad oocyte quality and impaired implantation of embryo are therefore considered to be the possible mechanisms for endometriosis-related infertility. Changes in cytokines and growth factors in endometrium, follicular fluid and peritoneal fluid have been suggested as the key players for inducing the above-mentioned phenomena [5]. Recently, several studies have shown that MUC4 could promote cell migration, change the endometrial environment and create weak points in the epithelium, thus facilitating the failure of embryo implantation [50,51]. The Carraway *et al*. [52] study also showed that embryo implantation was associated with downregulation of MUC4 expression in an animal model. In this study, women with a T allele in rs882605 had a lower risk of endometriosis-related infertility, whereas rs2688513 and rs2246901 SNPs did not show any association with the reproductive ability of patients. The rs882605 SNP locates in a putative functional loop within the VNTR domain of MUC4 that may control T-cell antigen presentation and the local immune responses. Our findings may support the view that the regulation of local immunity, rather than uncontrolled cell proliferation, in the endometrium may play a more important role in the development of endometriosis-related infertility.

Our study shows that the T/G genotype at rs882605, as compared with the T/T or G/G genotypes, is unique in patients with endometriosis. So far, our data cannot provide sufficient information to explain why the T/T genotype does not show higher risk of endometriosis than T/G. One reason could be the relatively small study group, while other possibilities could exist. For example, the SNPs analyzed are in tight LD with other unknown allele variants which have an opposite effect. In this case, only individuals with the T/G heterozygous genotype could be observed. Because this is a hospital-based study with a modest sample size, enrollment of a larger cohort based on a population approach could help to elucidate the functional role of MUC4 in endometriosis and the related infertility.

## Conclusions

In this study, we observed an association of *MUC4 *polymorphism with endometriosis development and endometriosis-related infertility in a Taiwanese population. However, the true mechanism of how MUC4 modulates the pathogenesis of endometriosis and infertility was not clearly understood. In addition, the risk SNPs pose for endometriosis stages and infertility differ, suggesting dissimilar molecular mechanisms for these clinical features. More detailed studies are needed to investigate the biochemical pathways regulated by MUC4 during the development of endometriosis.

## Competing interests

The authors declare that they have no competing interests.

## Authors' contributions

CYYC collected samples, carried out the clinical association study and drafted the manuscript. HWC participated in sample collection and the clinical association study. CMC carried out sample preparation and SNP analyses. CYL participated in SNP analyses. CPC participated in the clinical association study. CHL carried out statistical analyses. WYL carried out sample pretreatment and RNA extraction. JJCS carried out the experimental design and drafted the manuscript. FJT carried out the experimental design and revised the manuscript.

## Pre-publication history

The pre-publication history for this paper can be accessed here:

http://www.biomedcentral.com/1741-7015/9/19/prepub

## Supplementary Material

Additional file 1 **Supplementary Tables S1 to S3**. Click here for file

Additional file 2 **Supplementary Figures S1 and S2**. Click here for file
