# Supplementary material for: MUC4 gene polymorphisms associate with endometriosis development and endometriosis-related infertility
Source: BMC Med. 2011 Feb 24;9:19. doi: 10.1186/1741-7015-9-19 (PMC3052195; doi:10.1186/1741-7015-9-19)
Supplement: Additional file 1 — Supplementary Tables S1 to S3. [file 1741-7015-9-19-S1.DOC]

**Supplementary Table 1. Summary of the 142 controls and clinicopathological features of the 135 patients**

| **Clinical characteristic** | **No. (%) of patients** | | **No. (%) of control** | | |
| --- | --- | --- | --- | --- | --- |
| **Age strata** | total | n = 135 | total | | n = 142 |
| age <=30 | 43 | (31.85) | 49 | | (34.50) |
| age 31-40 | 51 | (37.78) | 56 | | (39.40) |
| age 41-50 | 39 | (28.89) | 35 | | (24.60) |
| age >=51 | 2 | (1.50) | 2 | | (1.40) |
| **Reproduction ability** |  |  |  | | |
| non-infertility | 97 | (71.85) |  | | |
| infertility | 20 | (14.82) |  | | |
| non sexual experience | 17 | (12.59) |  | | |
| data not available | 1 | (0.74) |  | | |
| **CA125 value** |  |  |  | | |
| CA125 <32 | 24 | (17.78) |  | | |
| CA125 <=32 | 55 | (40.74) |  | | |
| **Clinical stage** |  |  |  | | |
| mild (stage 1 or 2) | 8 | (5.93) |  | | |
| severe (stage 3 or 4) | 59 | (43.70) |  | | |
| **Pain score** |  |  |  | | |
| pain score 0-4 | 52 | (38.52) |  | | |
| pain score 5-10 | 71 | (52.59) |  |  | |

The average age in patient group is 33.62 , the average age in control group is 33.0.

**Supplementary Table 2. The allelic frequencies of the six SNPs in *MUC4* gene in International HapMap Project databank**

| dbSNP rs | allele frequency | | Amino acid position^a^ | ABI probe assay ID^b^ | chr location |
| --- | --- | --- | --- | --- | --- |
| rs882605 | G: 68% | T: 32% | Phe300Val | C_9712023_10 | 195517553 |
| rs1104760 | T: 68% | C: 32% | Thr377Ile | C_9712022_10 | 195517321 |
| rs2688513 | T: 69% | C: 31% | Pro4135Ser | C_26282126_10 | 195505664 |
| rs2246901 | T: 68% | G: 32% | Ala4693Ser | C_27058424_10 | 195489009 |
| rs2258447 | G: 71% | A: 29% | Glu5062Glu | C_27058459_10 | 195479256 |
| rs2291652 | T: 63% | C: 37% | Ile5152Ile | C_27058454_10 | 195477791 |

^a^The *MUC4* reference sequence in NCBI databank: NP_060876.4.

^b^The website of ABI probe assay ID search: <https://products.appliedbiosystems.com/ab/en/US/adirect/ab?cmd=ABGTKeywordSearch>

S**upplementary Table 3. The call rates for the six SNP probes tested in this study**

| SNP | group | subjects | detected subjects | call rate |
| --- | --- | --- | --- | --- |
| rs882605 | case | 135 | 130 | 96.3% |
|  | control | 142 | 142 | 100.0% |
| rs1104760 | case | 135 | 128 | 94.8% |
|  | control | 142 | 139 | 97.9% |
| rs2688513 | case | 135 | 133 | 98.5% |
|  | control | 142 | 142 | 100.0% |
| rs2246901 | case | 135 | 134 | 99.3% |
|  | control | 142 | 141 | 99.3% |
| rs2258447 | case | 135 | 134 | 99.3% |
|  | control | 142 | 142 | 100.0% |
| rs2291652 | case | 135 | 127 | 94.1% |
|  | control | 142 | 131 | 92.3% |
