# Supplementary material for: MUC4 gene polymorphisms associate with endometriosis development and endometriosis-related infertility
Source: BMC Med. 2011 Feb 24;9:19. doi: 10.1186/1741-7015-9-19 (PMC3052195; doi:10.1186/1741-7015-9-19)
Supplement: Additional file 2 — Supplementary Figures S1 and S2. [file 1741-7015-9-19-S2.DOC]

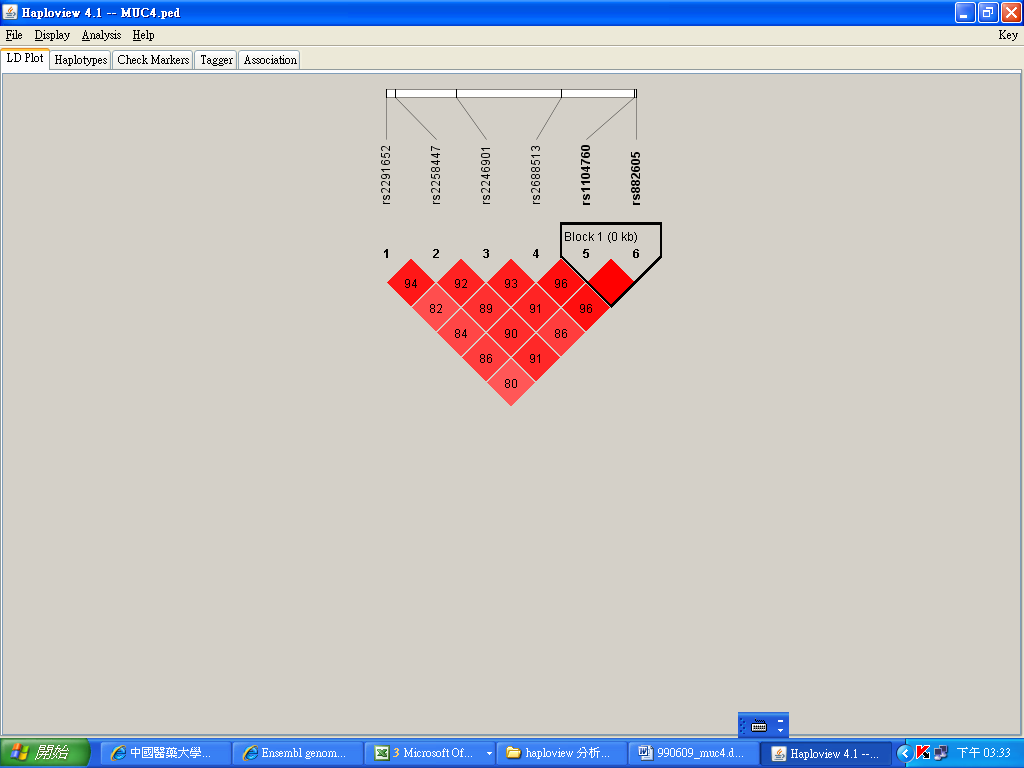


**Supplementary Figure 1 – The LD (linkage disequilibrium) blocks and correlation coefficients among the six SNPs in *MUC4* gene**

Haplotype blocks for the 142 control subjects and 135 endometriosis patients were constructed according to the confidence interval approach using Haploview software (see Methods). Red squares indicate statistically significant allelic association between a pair of SNPs, as measured by the D′ statistic. Darker shades of red indicate higher values of D′, up to a maximum of 1.


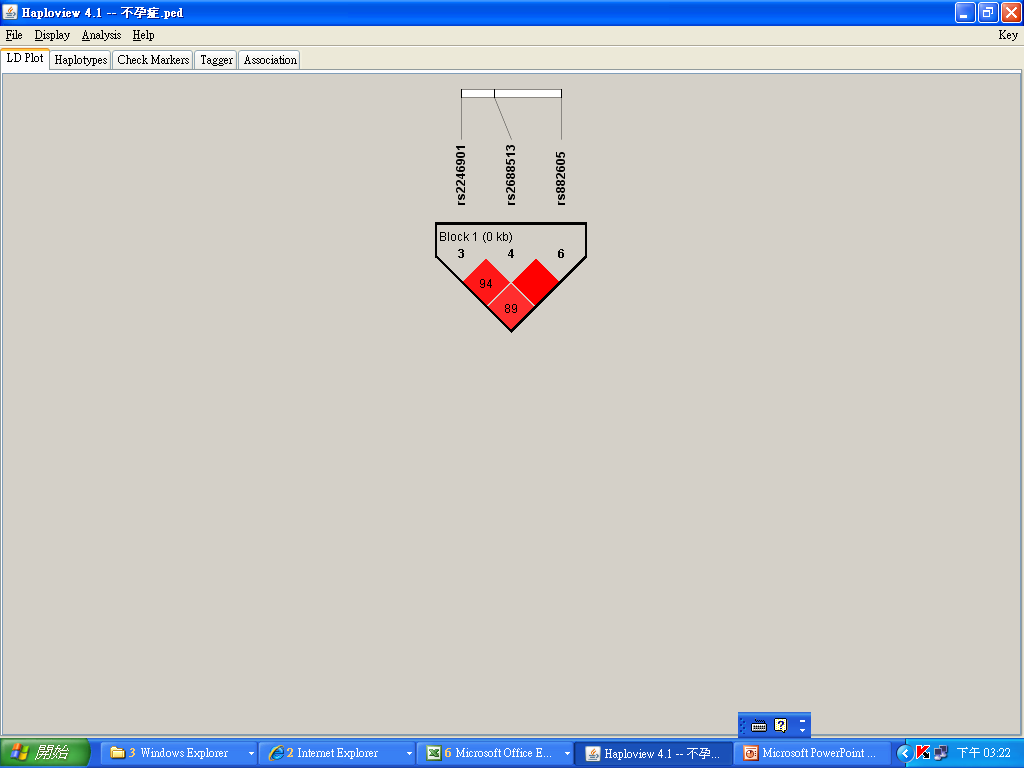


**Supplementary Figure 2 – The LD (linkage disequilibrium) blocks and correlation coefficients among the 3 SNPs in *MUC4* gene that associated with infertility in endometriosis patients**

Haplotype blocks for the 97 patients without infertility and 20 patients with infertility were constructed according to the confidence interval approach using Haploview software (see Methods). Red squares indicate statistically significant allelic association between a pair of SNPs, as measured by the D′ statistic. Darker shades of red indicate higher values of D′, up to a maximum of 1.
